# Low Mitochondrial DNA Copy Number is Associated With Adverse Clinical Outcomes in Peritoneal Dialysis Patients

**DOI:** 10.1097/MD.0000000000002717

**Published:** 2016-02-18

**Authors:** Chang-Yun Yoon, Jung Tak Park, Youn Kyung Kee, Seung Gyu Han, In Mee Han, Young Eun Kwon, Kyoung Sook Park, Mi Jung Lee, Seung Hyeok Han, Shin-Wook Kang, Tae-Hyun Yoo

**Affiliations:** From the Department of Internal Medicine (C-YY, JTP, YKK, SGH, IMH, YEK, KSP, MJL, SHH, S-WK, T-HY), Yonsei University College of Medicine; and Severance Biomedical Science Institute (S-WK, T-HY), Brain Korea 21 PLUS, Yonsei University College of Medicine, Seoul, Korea.

## Abstract

Mitochondrial dysfunction may play an important role in abnormal glucose metabolism and systemic inflammation. We aimed to investigate the relationship between mitochondrial DNA (mtDNA) copy number and clinical outcomes in peritoneal dialysis (PD) patients.

We recruited 120 prevalent PD patients and determined mtDNA copy number by PCR. Primary outcome was all-cause mortality, whereas secondary outcomes included cardiovascular events, technical PD failure, and incident malignancy. Cox proportional hazards analysis determined the independent association of mtDNA copy number with outcomes.

The mean patient age was 52.3 years; 42.5% were men. The mean log mtDNA copy number was 3.30 ± 0.50. During a follow-up period of 35.4 ± 19.3 months, all-cause mortality and secondary outcomes were observed in 20.0% and 59.2% of patients, respectively. Secondary outcomes were significantly lower in the highest mtDNA copy number group than in the lower groups. In multiple Cox analysis, the mtDNA copy number was not associated with all-cause mortality (lower two vs highest tertile: hazard ratio [HR] = 1.208, 95% confidence interval [CI] = 0.477–3.061). However, the highest tertile group was significantly associated with lower incidences of secondary outcomes (lower two vs highest tertile: HR [95% CI] = 0.494 [0.277–0.882]) after adjusting for confounding factors.

The decreased mtDNA copy number was significantly associated with adverse clinical outcomes in PD patients.

## Abstract 2

Supplemental Digital Content is available in the text

## INTRODUCTION

Mortality risks for end-stage renal disease (ESRD) patients are known to be higher than that for patients with other grave comorbidities such as cancer, stroke, or acute myocardial infarction.^1^ Extremely high mortality rates in ESRD patient are not fully explicable by traditional risk factors such as age, diabetes mellitus, smoking, hypertension, dyslipidemia, obesity, and family history.^2^ Therefore, recent investigations have attempted to explain these elevated mortality risks by evaluating the impact of so-called nontraditional risk factors such as uremia,^3^ insulin resistance,^4^ inflammation,^5^ and oxidative stress.^6^ Especially in patients maintaining PD, chronic exposure to high-glucose-containing peritoneal dialysis (PD) solution can induce many hazardous clinical outcomes including malnutrition, hypertriglyceridemia, poor glycemic control, and incident diabetes^7^ that are considered to be signs of mitochondrial injury.^8^

Mitochondria are ubiquitous organelles of eukaryotic systems that are essential for supplying cellular energy by the aerobic production of adenosine triphosphate via oxidative phosphorylation.^9^ Additionally, mitochondria are involved in numerous cellular tasks including regulation of metabolism, induction of reactive oxygen species (ROS) signaling, and apoptosis.^9^ Mitochondrial damage and consequent dysfunction are implicated in various cell metabolism-related human diseases such as cancer, neurodegenerative diseases, and diabetes.^10–12^ In particular, mitochondrial dysfunction in subjects who are older or have chronic illnesses is related to deletion of mitochondrial genes rather than mutations in mitochondrial DNA (mtDNA).^13^ Mitochondria are highly susceptible to oxidative stress and are a target of ROS as well producers of it. Recent data revealed that the mtDNA copy number reflects the level of mitochondrial biogenesis as well as the amount of mtDNA. The low mtDNA copy number is associated with aging and various human diseases, including diabetes, cardiomyopathy, and cancer.^14^

Recent studies reported that the mtDNA copy number was significantly associated with clinical outcome in dialysis patients.^15,16^ A low mtDNA copy number correlates with increased oxidative stress and higher mortality rates in patients undergoing dialysis. In a uremic rat model, the mtDNA copy number was also significantly decreased compared to control rats, and was associated with decreased renal function.^17^ Such clinical and experimental studies suggest that the mtDNA copy number could be a potential biomarker for clinical outcomes in dialysis patients as well as the general population. Therefore, we aimed to test this hypothesis by investigating the association between the mtDNA copy number and clinical outcomes in patients undergoing PD.

## METHODS

### Ethics Statement

The study was carried out in accordance with the Declaration of Helsinki and approved by the Institutional Review Board of Yonsei University Health System (YUHS) Clinical Trial Center. We obtained informed written consent from all participants involved.

### Subjects

**FIGURE 1:**
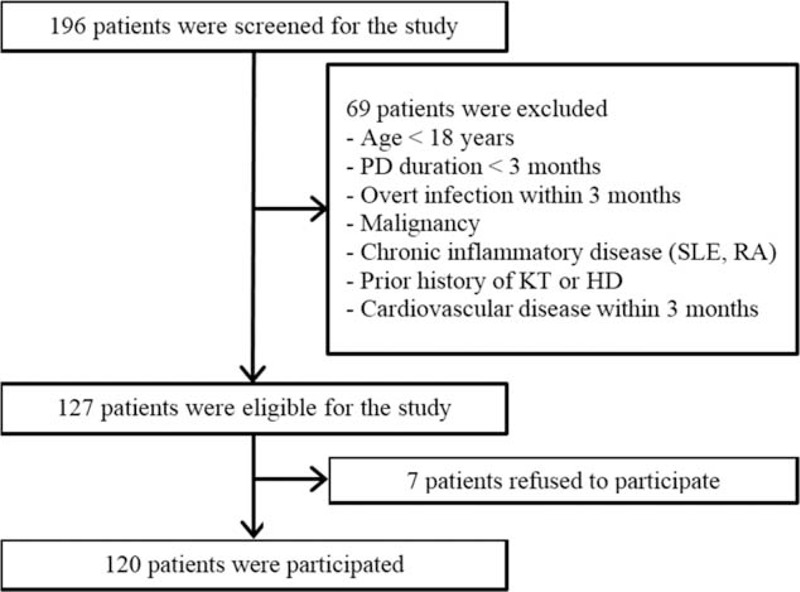
Flow diagram of the study. HD = hemodialysis, KT = kidney transplantation, PD = peritoneal dialysis, RA = rheumatoid arthritis, SLE = systemic lupus erythematosus.

The study population was comprised of participants in a prospective cohort that included prevalent PD patients at the YUHS. The study was designed to investigate cardiovascular risk and mortality in PD patients.^18^ All consecutive ESRD patients >18 years of age who underwent PD for >3 months at YUHS were initially screened for enrollment between February 2010 and Dec 2011. Patients were excluded if they had histories of overt infection or malignancy, or had another chronic inflammatory disease such as rheumatoid arthritis or systemic lupus erythematosus, within 3 months of enrollment. Patients with a history of kidney transplantation, hemodialysis for >3 months before PD, or cardiovascular disease within the prior 3 months were also excluded. Ultimately, 120 prevalent PD patients were included (Figure 1).

A senior nursing clinician obtained demographic data via interviews. Demographic and clinical data recorded at study entry included age, sex, and PD duration. Weight, height, and biochemical data were measured at study enrollment. The body mass index was calculated as weight/height (kg/m^2^). Diabetes mellitus and hypertension were defined as described previously.^19,20^

### Laboratory Data Collection

The blood samples were obtained from study subjects after overnight fasting for 12 h, and plasma was extracted and frozen at −70°C before experiment. The laboratory values including hemoglobin, albumin, lipid profiles, calcium, phosphorus, and high-sensitivity C-reactive protein (hs-CRP) were measured at baseline. Fasting plasma glucose was determined by the glucose oxidase method. Lipid profiles including total cholesterol, high-density lipoprotein cholesterol (HDL-C), low-density lipoprotein cholesterol, and triglyceride were measured by enzymatic colorimetry using an autoanalyser (Hitachi 7150, Hitachi Ltd, Tokyo, Japan), and hs-CRP was determined by a latex-enhanced immunonephelometric method using a BNII analyzer (Dade Behring, Newark, DE). Insulin resistance was assessed by using the homeostatic model assessment-insulin resistance (HOMA-IR) equation as follows: HOMA-IR = (fasting insulin in microunit per liter *×* fasting plasma glucose in millimoles per liter/22.5).^21^ Plasma adiponectin (B-Bridge International, Sunnyvale, CA) and leptin (R&D Systems, Minneapolis, MN) were measured using enzyme-linked immunosorbent assays.

### Definition of Malnutrition Inflammation Score

The malnutrition inflammation score (MIS) was calculated as described previously.^22^ The Subjective Global Assessment (SGA) of nutritional state was assessed by history and physical examination.^23^ The Dialysis Malnutrition Score (DMS),^24^ which incorporates 7 conventional SGA components (weight change, dietary intake, gastrointestinal symptoms, functional capacity, comorbidity, subcutaneous fat, and signs of muscle wasting), was included. To attempt to produce a more comprehensive and quantitative scoring system, 3 new items were added to the DMS criteria: body mass index, serum albumin levels, and total iron-binding capacity. The MIS has 10 components ranging from 0 (normal) to 30 (severely malnourished); a higher score reflects a more severe degree of malnutrition and inflammation.^22^

### Quantification of mtDNA Copy Number

Nucleic acids were extracted from white blood cells using a previously described method.^25^ An assay based on real-time quantitative PCR was used for the mtDNA copy number quantification using SYBR green as a fluorescent dye (Invitrogen, Buenos Aires, Argentina). Quantification of mtDNA copy number was performed as described previously.^8,26^ Briefly, DNA was extracted from a 1 mL peripheral whole blood sample using the QIAamp Tissue Kit 250 (Qiagen Inc, Valencia, CA), according to the manufacturer's instructions. The mtDNA copy number was expressed as a relative ratio between quantitative PCR measured mtDNA gene copy number and a simultaneous measurement of nuclear DNA copy number. To assess the mtDNA gene copy number, a 120-bp-long mtDNA fragment within the mitochondrial *ND1* gene was amplified. The *ND1* forward primer used was 5′CCCTAAAACCCGCCACATCT3′, and reverse primer was 5′GAGCGATGGTGAGAGCTAAGGT3′. The lipoprotein lipase gene was amplified to assess the nuclear DNA copy number using the primers forward 5′CGAGTCGTCTTTCTCCTGATGAT′ and reverse 5′TTCTGGATTCCAATGCTTCGA′. Real-time PCR was conducted in a total of 20-μL volume, 10 μL of SYBR Green Master Mix (Applied Biosystems), 5 μL of DNA, and 5 pM each of primers. The PCR cycles were conditioned by initial heating at 95°C for 9 minutes; 35 cycles of denaturation at 94.5°C for 30 s, annealing at 60°C for 30 s, and extension at 72°C for 1 minute; and final extension at 72°C for 7 minutes. Triplicate analysis was performed in each sample. After the reaction of PCR, the temperature was increased to 95°C at a rate of 2°C/minute to make a melting curve.^27^ The ratio of mtDNA/nuclear DNA was calculated as 2^ΔCT^, where ΔCT is CT_LDL_ − CT_ND1_. Intra-assay and inter-assay coefficients of variation of mtDNA copy number were 4.7% (range 1.8–6.7) and 5.9% (range 3.7–9.1), respectively.

### Assessment of Dialysis Adequacy and Lean Body Mass Using Creatinine Kinetics

Urea kinetics studies were conducted based on a 24-hour collection of dialysate and urine at the time of study enrollment. Kt/V urea was determined from the total loss of urea nitrogen in spent dialysate using PD Adequest 2.0 for Windows software (Baxter Healthcare, Deerfield, IL). Lean body mass was estimated by creatinine kinetics, and the percentage lean body mass was calculated as lean body mass normalized to dry weight.

### Follow-Up and Endpoints

Patients were seen for follow-up at 3-month intervals through July 1, 2015. All deaths and hospitalizations were documented in a database from which all events were retrieved. Primary outcome was defined as all-cause mortality. Secondary outcomes were defined as major cardiovascular events, PD failure, or incident malignancy. Cardiovascular events were defined as death or hospitalization from an acute coronary syndrome and stable angina requiring coronary revascularization or coronary bypass surgery. Cerebrovascular events and peripheral vascular disease were also deemed major cardiovascular events. Cerebrovascular events were defined as hospitalization or visit to the emergency department for a transient ischemic attack, ischemic stroke, or carotid endarterectomy. Peripheral vascular disease was defined as ischemic limb loss and/or ulceration or peripheral revascularization procedure. PD failure referred to a technical failure of PD. Incident malignancy was defined as malignancy that was confirmed by pathologic or radiological diagnosis regardless of primary origin or stage. Subjects lost to follow-up and kidney transplantation recipients were censored in the final analysis.

### Statistical Analysis

The means and standard deviations were used to indicate continuous variables, and frequencies and percentages were estimated for categorical data. The Kolmogorov–Smirnov test was performed to confirm the normality of distribution. The trichotomized mtDNA copy number groups were compared by ANOVA for continuous variables and the trend test or Fisher's exact test for categorical variables. To test the significant factors associated with mtDNA copy number, univariate and multivariate linear regression analyses were conducted. Regarding the distribution of variables, triglyceride, intact-parathyroid hormone, ferritin, leptin, leptin–adiponectin ratio, hs-CRP, HOMA-IR, and mtDNA copy number were transformed to natural logarithmic values for linear regression analysis. Variables that had *P* values <0.1 on univariate linear regression analysis were included in multivariate linear regression analysis. Comparisons between the highest mtDNA copy number tertile and the lower two mtDNA copy number tertiles were performed by Kaplan–Meier analysis and a log-rank test. The independent predictive role of mtDNA copy number in clinical outcomes was determined by Cox proportional hazard analysis, which included significant variables in the comparison of baseline characteristics and linear regression analysis. Statistical analysis was performed with SPSS for Windows version 20.0 (IBM SPSS Inc, Chicago, IL). *P* values of <0.05 were considered significant.

## RESULTS

### Baseline Characteristics According to the mtDNA Copy Number

The baseline characteristics are shown in Table 1. The mean age of the study population was 52.3 ± 12.0 years; 51 (42.5%) patients were men. The mean duration of PD was 88.5 ± 49.3 months, and the mean log mtDNA copy number was 3.30 ± 0.50. Patients were divided into 3 groups according to their mtDNA copy number. Previous history of coronary artery disease (12.5% vs 7.5% vs 0%, *P* = 0.04) and MIS (6.3 ± 2.2 vs 6.8 ± 1.7 vs 3.9 ± 1.4 respectively, *P* < 0.001) were significantly lower in the highest mtDNA copy number group compared to the lower and middle groups, respectively.

**TABLE 1:**
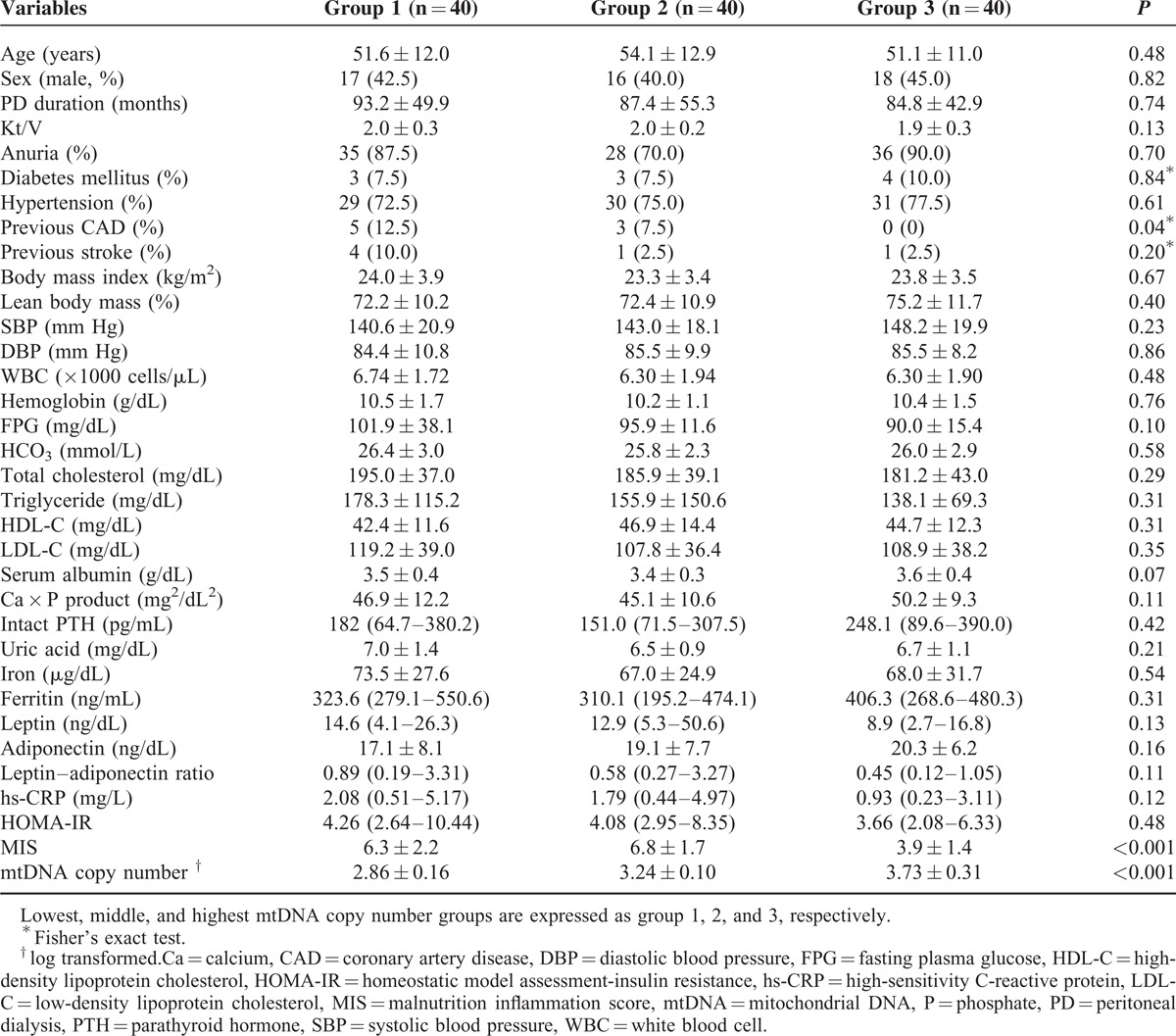
Baseline Characteristics of Subjects According to the mtDNA Copy Number

### Composites of Secondary Outcome Were Significantly Lower in the Highest mtDNA Copy Number Group

During a mean follow-up duration of 35.4 ± 19.3 months, all-cause mortality and secondary outcomes were observed in 24 (20.0%) and 71 (59.2%) patients, respectively (Table 2). There were no significant differences in all-cause mortality in any of the trichotomized mtDNA copy number groups (25.0% vs 15.0% vs 20.0% respectively, *P* = 0.68). Meanwhile, composites of secondary outcome were significantly lower in the highest mtDNA copy number group compared to the lower or middle mtDNA copy number groups (75.0% vs 62.5% vs 40.0% respectively, *P* = 0.002). Among secondary outcomes, PD failure (55.0% vs 55.0% vs 27.5% respectively, *P* = 0.02) and incident malignancy (30.0% vs 20.0% vs 10.0% respectively, *P* = 0.04) were significantly lower in the highest tertile mtDNA copy number group. As for incident malignancies, urogenital cancer was the most frequent (8 renal cell carcinoma, 2 ureter cancers, and 1 bladder cancer), followed by gastrointestinal cancer (2 stomach cancers, 2 colorectal cancers, and 1 hepatocellular carcinoma), breast and gynecologic cancers (ovarian, cervical, and endometrial cancer, respectively), and thyroid cancer. There was no significant difference in cardiovascular events among the groups (15.0% vs 17.5% vs 7.5% respectively, *P* = 0.42).

**TABLE 2:**
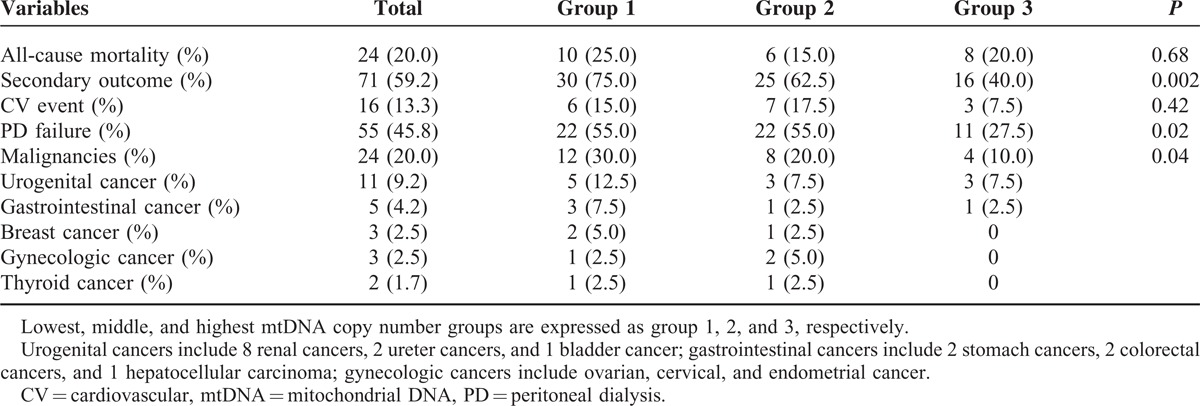
Clinical Outcome According to mtDNA Copy Number

### Association of Clinical and Biochemical Variables With mtDNA Copy Number

Linear regression analyses were performed to investigate the association between clinical/biochemical variables and log mtDNA copy number. On univariate analysis, previous coronary artery disease (β = −0.622, *P* = 0.007), white blood cell (per 1000 cells/μL, β = −0.065, *P* = 0.04), fasting plasma glucose (β = −0.004, *P* = 0.02), log hs-CRP (β = −0.082, *P* = 0.03), log HOMA-IR (β = −0.130, *P* = 0.03), and MIS (β = −0.088, *P* = 0.02) were negatively correlated with mtDNA copy number, whereas adiponectin (β = 0.016, *P* = 0.03) was positively correlated (data not shown). Multivariate linear regression analysis was also performed to clarify the independent association of variables with log mtDNA copy number (Table 3). The analysis indicated that log mtDNA copy number was independently associated with log hs-CRP (β = −0.085, *P* = 0.04) and MIS (β = −0.072, *P* = 0.03).

**TABLE 3:**
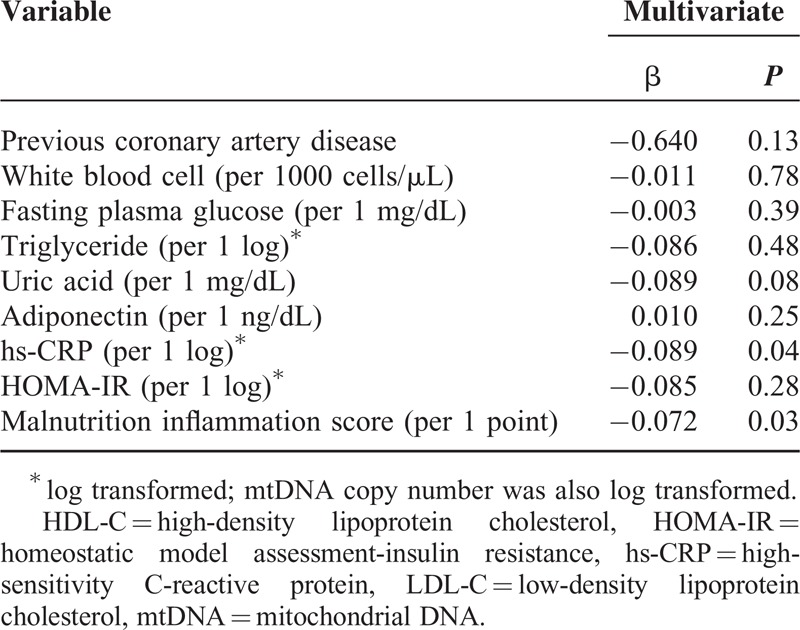
Multivariate Linear Regression Analysis for the Association of mtDNA Copy Number With Clinical and Biochemical Variables

### Low mtDNA Copy Number Is an Independent Risk Factor for Adverse Clinical Outcomes in PD Patients

**FIGURE 2:**
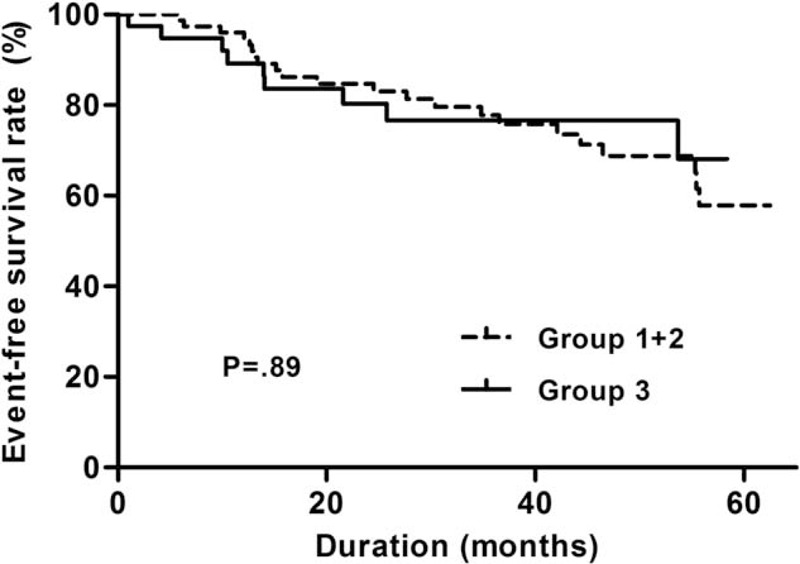
Kaplan–Meier plots for all-cause mortality-free survival between the lower two and the highest mtDNA copy number tertile groups. Lowest, middle, and highest mtDNA copy number groups are expressed as group 1, 2, and 3, respectively. mtDNA = mitochondrial DNA.

**FIGURE 3:**
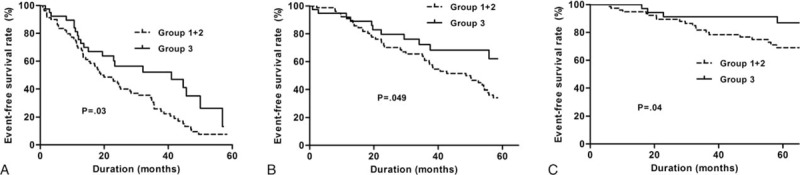
Kaplan–Meier plots for composites of secondary outcomes-free survival between the lower two and the highest mtDNA copy number tertile groups. Composites of secondary outcomes included cardiovascular events, peritoneal dialysis failure, and incident malignancies. Lowest, middle, and highest mtDNA copy number groups are expressed as group 1, 2, and 3, respectively. (B) Kaplan–Meier plots for peritoneal dialysis failure-free survival between the lower two and the highest mtDNA copy number tertile group. Lowest, middle, and highest mtDNA copy number groups are expressed as group 1, 2, and 3, respectively. (C) Kaplan–Meier plots comparing incident malignancy-free survival between the lower two and the highest mtDNA copy number tertile groups. Lowest, middle, and highest mtDNA copy number groups are expressed as group 1, 2, and 3, respectively. mtDNA = mitochondrial DNA.

Multivariate Cox proportional hazard analysis was performed to investigate the prognostic role of mtDNA copy number in all-cause mortality, cardiovascular events, PD failures, and incident malignancies in PD patients (Table 4). The mtDNA copy number was not significantly associated with all-cause mortality (lower two groups vs highest: hazard ratio [HR] = 1.21, confidence interval [CI] = 0.477–3.061, *P* = 0.69) after adjustment for age, sex, PD duration, previous history of coronary artery disease, serum albumin, hs-CRP, and MIS. However, the composites of secondary outcome were significantly decreased in the highest mtDNA copy number group compared to the lower two mtDNA copy number groups after adjustment for the same confounding factors (lower two groups vs highest: HR [CI] = 0.494 [0.277–0.882], *P* = 0.02). Kaplan–Meier plots showed that all-cause mortality was not significantly different between the mtDNA copy number groups (log-rank test; *P* = 0.89; Figure 2). However, the composites of secondary outcomes were significantly lower in the highest mtDNA copy number group compared to the lower two mtDNA copy number groups (*P* = 0.03). Moreover, event rates of PD failure (*P* = 0.049) and incident malignancies (*P* = 0.04) were significantly lower in the highest mtDNA copy number group compared to the lower two groups (Figure 3).

**TABLE 4:**
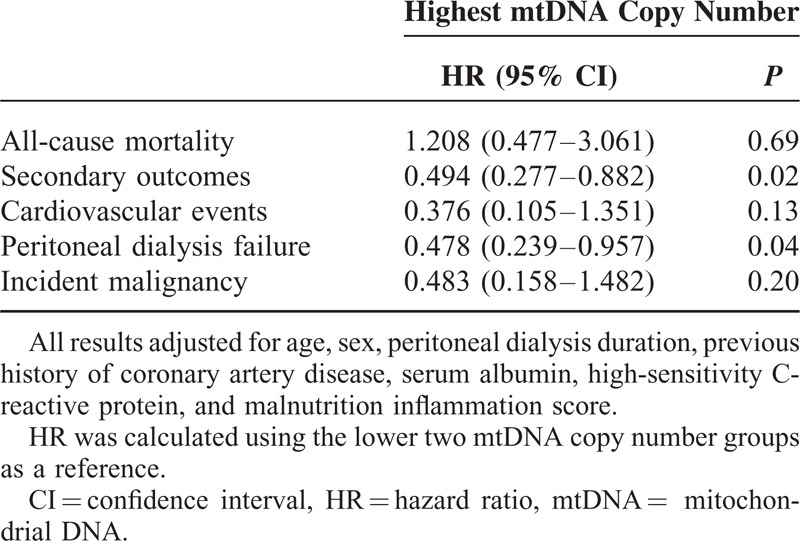
Multivariate Cox Proportional Hazard Regression Analysis for the Association of mtDNA Copy Number With Clinical Outcomes

## DISCUSSION

Our data showed that the mtDNA copy number is significantly associated with inflammatory or nutritional markers such as hs-CRP and MIS in PD patients. Furthermore, the mtDNA copy number was independently associated with adverse clinical outcomes, including PD failure and incident malignancy.

Many studies revealed that mitochondria have an essential role in the pathophysiology of diverse diseases and aging.^13,16,28^ Initially, genetic mutations in mitochondria had been observed in rare inherited disorders; however, recent studies reported that mitochondrial dysfunction and low mtDNA copy number have been implicated in the pathogenesis of chronic metabolic illnesses such as diabetes^11^ and dementia.^29^ Although the mechanisms surrounding decreased mtDNA copy number on several diseases are still not fully understood, several studies point to oxidative stress, inflammation, and malnutrition as highly probable factors.^30–32^ As a modest increase in oxidative stress and chronic inflammation was frequently observed in chronic kidney disease patients, including those on dialysis,^6^ chronic exposure to uremia has also been proposed to play a role in inducing mitochondrial deficiency and consequent dysfunction. In addition, protein-energy wasting is commonly observed and closely linked to poor clinical outcomes in ESRD patients.^33^ Especially, patients treated with PD are highly vulnerable to malnutrition caused by inadequate protein intake combined with persistent protein loss through the peritoneal membrane.^34^ Moreover, chronic inflammation and pro-inflammatory cytokines cause malnutrition by increasing protein hydrolysis.^35^ The present study also demonstrated that the mtDNA copy number is closely linked to hs-CRP as an inflammatory marker and to MIS as a nutritional marker. These findings suggest that mitochondrial abnormalities could be potential pathophysiologic mechanisms inducing malnutrition-inflammation-cachexia syndrome in PD patients.

In the present study, the low mtDNA copy number was associated with a higher technical failure rate in prevalent PD patients. It is unclear why a low mtDNA copy number results in technical failure. PD peritonitis is the main cause of catheter removal in PD patients,^36^ and recurrent or refractory PD peritonitis was the single most common reason for transfer to hemodialysis in the study subjects. Moreover, the number of peritonitis-related technical failures was greater in the lower two mtDNA copy number groups compared to the highest mtDNA copy number group (68.2% vs 54.6%, respectively). Recent reports demonstrated that mitochondria participate in innate immunity and are involved in immune response to bacterial and viral infection.^37^ It is assumed that host immunity is modulated by mitochondria, and mitochondrial dysfunction increases susceptibility to bacterial or viral infection. On the other hand, risk factors related to PD peritonitis include diabetes and hypoalbuminemia as markers for malnutrition and residual renal function.^38,39^ As mentioned above, the mtDNA copy number is closely associated with MIS. We surmised that malnutrition and susceptibility to infection are related to the low mtDNA copy number and might increase the risk of PD peritonitis in our subjects. Therefore, the increased peritonitis rate may partially account for a higher technical failure rate in the low mtDNA copy number groups.

Although the present study showed that the low mtDNA copy number as an independent predictor of adverse clinical outcomes in PD patients, evidence to suggest that the mtDNA copy number is a direct indicator of adverse clinical outcomes in PD remains weak. However, several previous studies showed that a low mtDNA copy number is predictive of all-cause mortality or incident cardiovascular events in highly morbid populations such as those with heart failure and ESRD.^16,40^ Furthermore, the mtDNA copy number is closely related to the nontraditional risk factor such as inflammation.^41^ It is well known that oxidative stress is closely related to chronic inflammation and that mitochondria are highly susceptible to oxidative stress. We speculated that the low mtDNA copy number, which is a consequence of chronic inflammation and oxidative stress, was associated with poor clinical outcomes in the present study.

The mtDNA copy number and mitochondrial dysfunction have also been reported to be closely related to the development of malignancy in the general population.^42^ Furthermore, dialysis patients have a higher risk of developing cancer.^43^ Although mitochondrial dysfunction may be prevalent in ESRD patients, the association between cancer risk and mitochondrial dysfunction is still elusive. The relationship between the low mtDNA copy number and risk of malignancy in this study suggests that the low mtDNA copy number and consequent mitochondrial dysfunction could increase the risks of developing malignancies in dialysis patients.

There are several limitations to our study. First, the study subjects were relatively small in number and were undergoing prevalent PD at a single center, possibly causing selection bias. Additionally, the significant differences found in adverse clinical outcomes were observed when comparing the highest tertile to the lowest or lower two mtDNA copy number tertiles and not in a stepwise manner (Supplementary Figures 1 and 2). These results may be due to the small population size. Moreover, there exists the possibility that the threshold mtDNA copy number control, which was proposed by Laura et al, might partly influence these results.^14^ There is a pathologic threshold level of mtDNA copy number that needs to be maintained for healthy homeostasis, and the reduced mtDNA copy number below the threshold in PD patients may lead to various adverse clinical outcomes. However, the exact triggering mechanism is still obscure; therefore, larger scale studies are required to confirm our findings. The absence of subsequent mtDNA copy number measurements during follow-up was another limitation in this study. The mtDNA copy number of the study population may have changed during the follow-up period, and obtaining average levels over time would have added reliability. Although we did not check mtDNA copy number serially, we only enrolled patients on PD who were relatively stable. As the study was observational rather than interventional, and single measurements of the mtDNA copy number at baseline were used for analysis, the causal relationship between mtDNA copy number and clinical outcomes has not been clarified.

In conclusion, we demonstrated that the low mtDNA copy number is significantly associated with adverse clinical outcomes in maintenance PD patients. It is also significantly associated with markers of systemic inflammation and malnutrition. These findings suggest that the mtDNA copy number could be helpful in predicting clinical outcomes in PD patients.

## Supplementary Material

Supplemental Digital Content
