# Supplementary material for: Low Mitochondrial DNA Copy Number is Associated With Adverse Clinical Outcomes in Peritoneal Dialysis Patients
Source: Medicine (Baltimore). 2016 Feb 18;95(7):e2717. doi: 10.1097/MD.0000000000002717 (PMC4998611; doi:10.1097/MD.0000000000002717)

***Supplementary Figure 1.*** Kaplan-Meier plots for all-cause mortality-free survival among trichotomized mtDNA copy number groups. Lowest, middle, and highest mtDNA copy number groups are expressed as group 1,2, and 3, respectively. *Abbreviation:* mtDNA, mitochondrial DNA

**
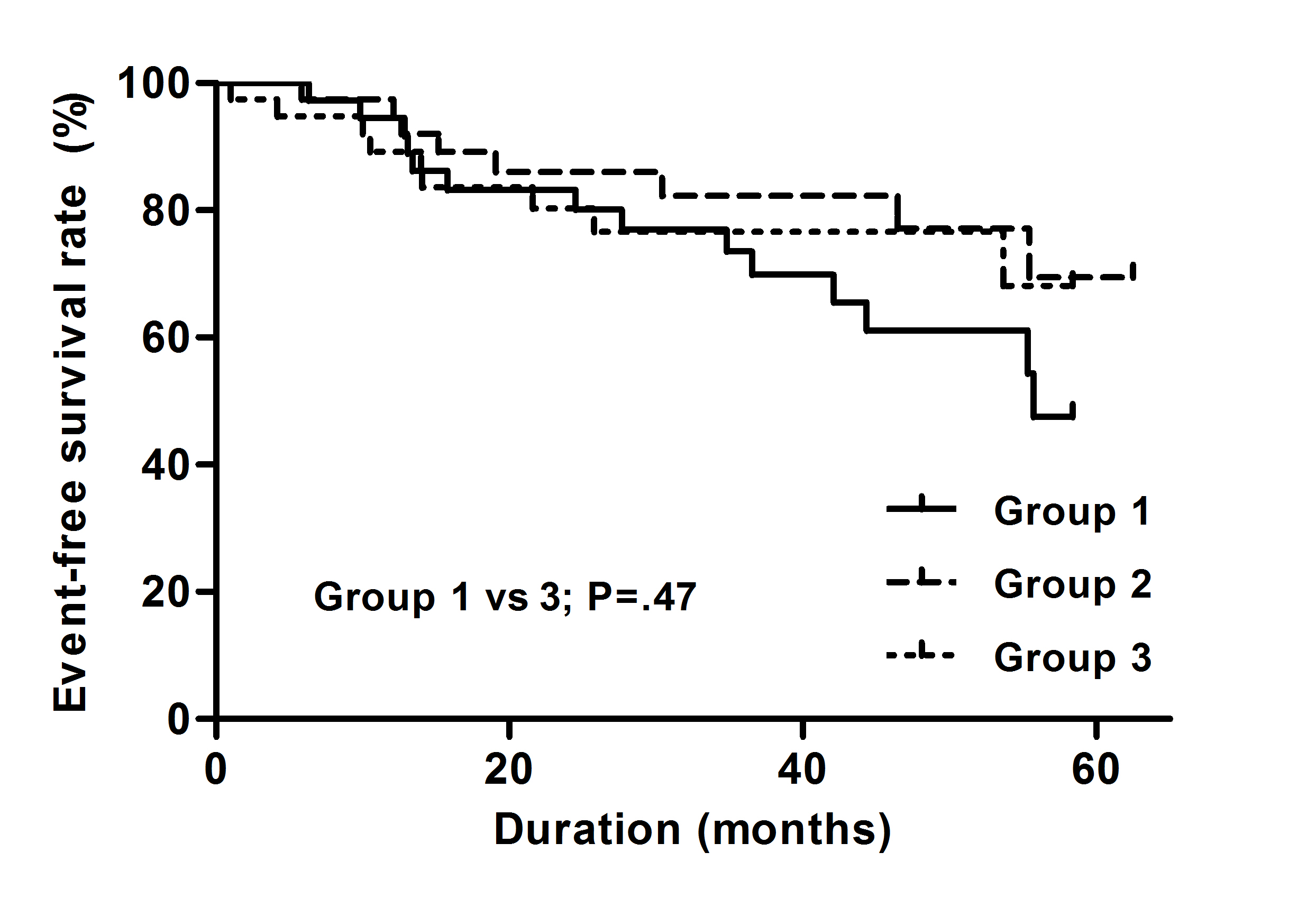
**

***Supplementary Figure 2-A.*** Kaplan-Meier plots for composites of secondary outcome-free survival among trichotomized mtDNA copy number groups. Composites of secondary outcomes included cardiovascular events, peritoneal dialysis failure, and incident malignancies. Lowest, middle, and highest mtDNA copy number groups are expressed as group 1,2, and 3, respectively. *Abbreviation:* mtDNA, mitochondrial DNA

**
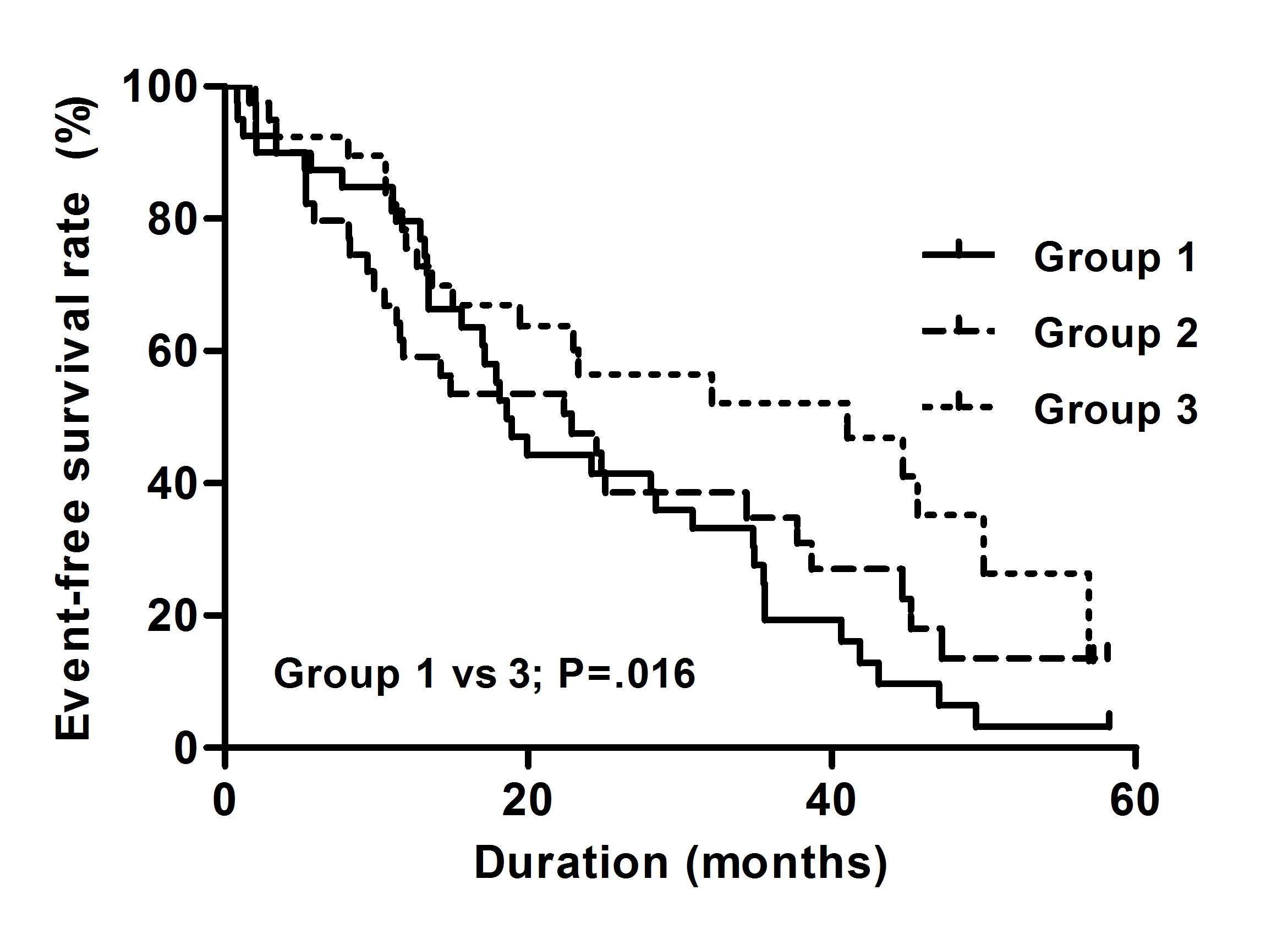
**

***Supplementary Figure 2-B.*** Kaplan-Meier plots for peritoneal dialysis failure-free survival among trichotomized mtDNA copy number groups. Lowest, middle, and highest mtDNA copy number groups are expressed as group 1,2, and 3, respectively. *Abbreviation:* mtDNA, mitochondrial DNA


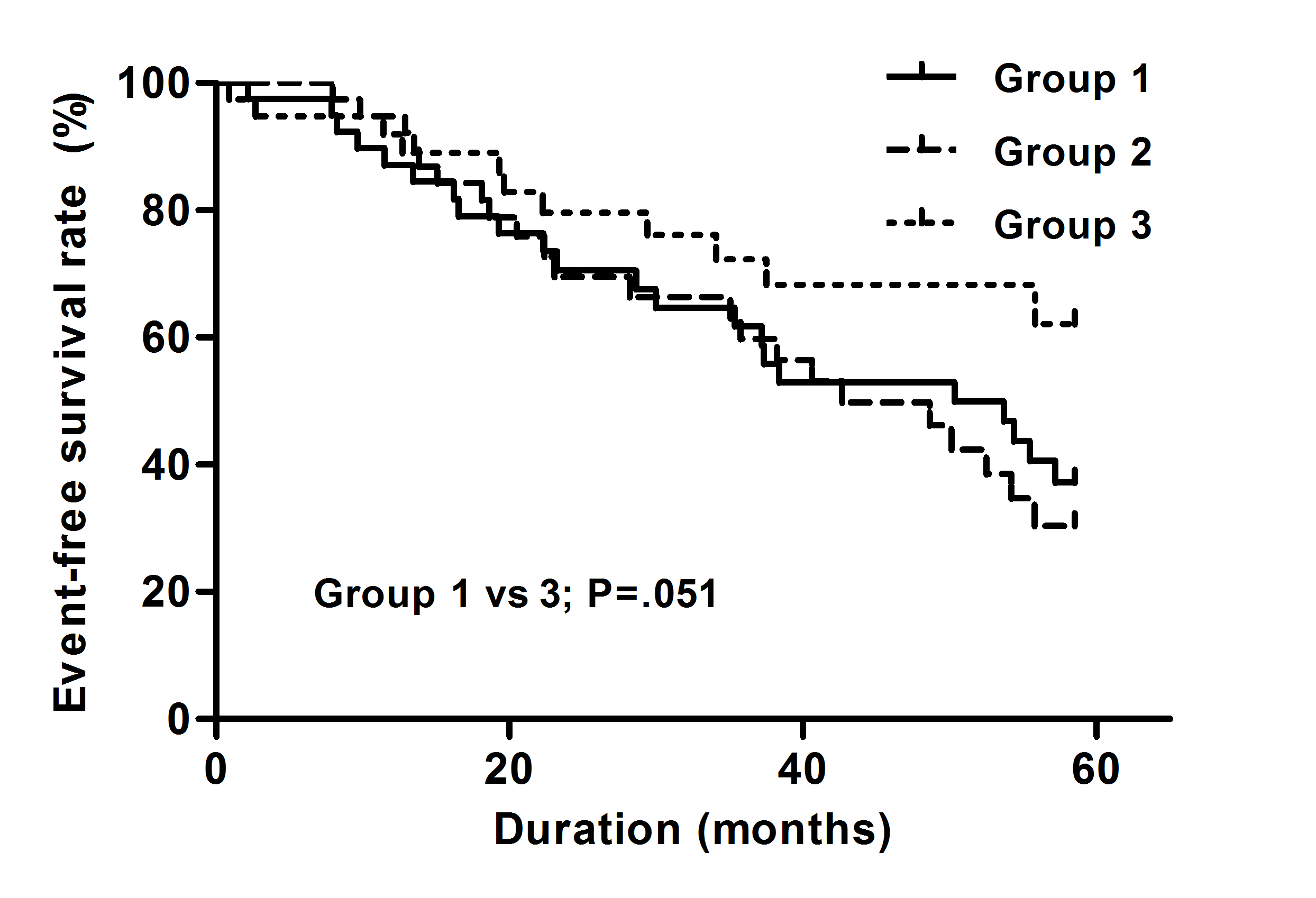


***Supplementary Figure 2-C.*** Kaplan-Meier plots comparing incident malignancy-free survival among trichotomized mtDNA copy number groups. Lowest, middle, and highest mtDNA copy number groups are expressed as group 1,2, and 3, respectively. *Abbreviation:* mtDNA, mitochondrial DNA


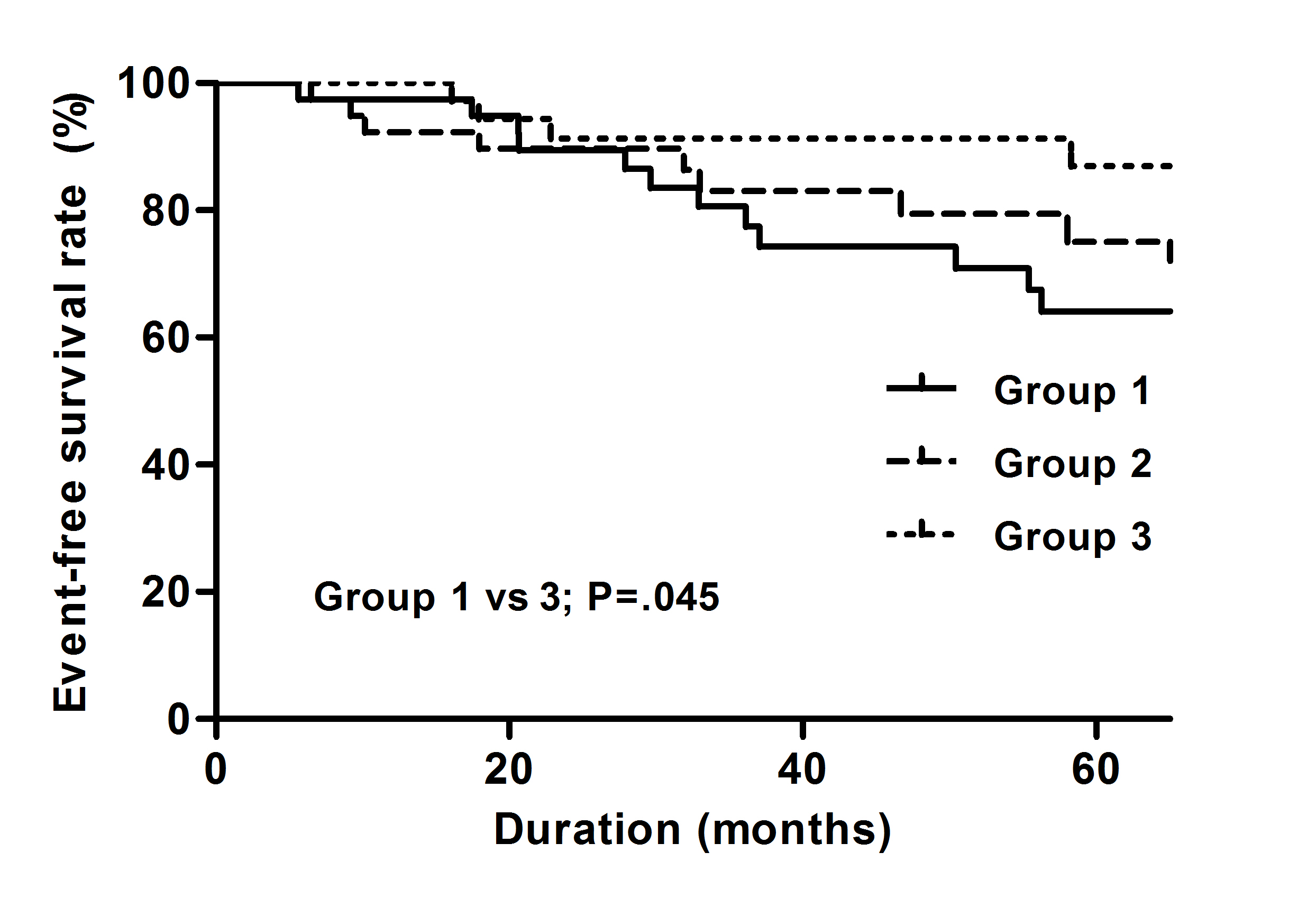

Supplement: Supplemental Digital Content [file medi-95-e2717-s001.doc]
